# Description of *Onthophagus humboldti* and *Uroxys bonplandi*, two new scarab beetles (Coleoptera, Scarabaeidae, Scarabaeinae) from Costa Rica, with notes on tropical mountain brachyptery and endemicity

**DOI:** 10.3897/zookeys.881.38026

**Published:** 2019-10-17

**Authors:** Bert Kohlmann, Ángel Solís, Guillermo E. Alvarado

**Affiliations:** 1 Universidad EARTH, AP 4442–1000, San José, Costa Rica Universidad EARTH San José Costa Rica; 2 Museo Nacional de Costa Rica, AP 749–1000, San José, Costa Rica Museo Nacional de Costa Rica San José Costa Rica; 3 Centro de Investigaciones en Ciencias Geológicas, Universidad de Costa Rica, San José, Costa Rica Universidad de Costa Rica San José Costa Rica

**Keywords:** Biogeography, boreotropical distribution, Cordillera de Talamanca, extreme brachyptery, refugia, Last Glacial Maximum, mitochondrial DNA, paleogeography

## Abstract

Two new endemic species of scarab beetles are described from Costa Rica, *Onthophagus humboldti***sp. nov**. and *Uroxys bonplandi***sp. nov.***Onthophagus humboldti***sp. nov.** is also the tenth brachypterous *Onthophagus* species to be described worldwide, representing also a case of extreme brachyptery in Onthophagini. Illustrations for both new species, as well as marking differences with closely related species are included. Maps showing the distribution of the new species, as well as the distribution of brachypterous and endemic scarab-beetle species for Costa Rica are presented and discussed. The Cordillera de Talamanca represents an area where Scarabaeinae (four genera) show very high known levels of brachypterism in Mesoamerica. A reconstruction of the montane environment in the Cordillera de Talamanca during the Last Glacial Maximum (~24 ka) is analyzed, in order to try to understand a possible historical biogeography model that might promote high levels of brachypterism in scarab-beetles. The present study supports previous proposals that brachyptery is correlated with stable environments associated with deeply incised valleys. Tropical mountain ranges are also identified as having more endemics than lowland rain forests, contradicting accepted wisdom. Lastly, a mitochondrial DNA analysis supports the existence of the *Onthophagus dicranius* and the *O. clypeatus* species-groups as two well-defined and closely related branches.

*Notre imagination n’est frappée que par ce qui est grand; mais l’amoureux de la philosophie naturelle devrait également réfléchir aux petites choses.*

*Alexander von Humboldt*

“*Voyage aux régions équinoxiales du nouveau continent”, 1814*

## Introduction

During the last 27 years, a concerted effort has been undertaken by the first two authors in order to study the scarab beetles (Scarabaeidae: Scarabaeinae) of Costa Rica. The detailed evaluation of the specimens of this survey has yielded many new species. Currently, Scarabaeinae in Costa Rica are represented by seven tribes and 28 genera (Solís and Kohlmann 2012). This study is elevating the number of known scarab species from 182 to 184. These numbers will certainly increase in the future, as new species and new country records are discovered. We consider Costa Rica to be one of the best-known tropical countries in relation to the systematics and distribution of scarab beetles.

The discovery of these two new species of scarab beetles, one brachypterous (*Onthophagus*) and both of them endemic to the country, bring to the forefront questions regarding the existence of such interesting phenomena as brachyptery and endemicity. These two mechanisms seem to be concentrated in the mountainous areas in Costa Rica. Using these new species as a model, an attempt is made to try to understand the existence of these two processes in the mountains of the tropics.

Considering the small area that Costa Rica occupies (51,100 km^2^), it displays a great number of brachypterous scarab-beetle species (7) from four genera (*Ateuchus*, *Canthidium*, *Cryptocanthon*, *Onthophagus).* This represents a figure of 0.014 brachypterous species / 100 km^2^. One can compare this number with the state of Oaxaca in Mexico, an area arguably similar to Costa Rica in extension (93,952 km^2^) and biogeography/ecology. This Mexican state reports four brachypterous scarabaeines from two genera (*Canthidium* and *Onthophagus*) (Kohlmann in press). This would account for 0.004 brachypterous species / 100 km^2^. These lofty numbers certainly beg the question about a possible brachyptery-generating mechanism. In order to try to explain this situation, a paleoclimatic/paleogeographic model is here developed for the Cordillera de Talamanca (Talamanca range) in Costa Rica, spanning to the Last Glacial Maximum (LGM, ~25–23 ka).

Recent biogeographical studies of these scarab beetles in Costa Rica (Kohlmann 2011; Kohlmann et al. 2007, 2010) have detected new areas of high endemicity and species richness. These studies have highlighted the existence and importance of tropical mountains as areas of high biodiversity and endemicity, debunking the commonly held belief that lowland tropical rain forests reign supreme on these accounts (Fogden and Fogden 1997; Valerio 2006). Obando´s (2002) and Kohlmann´s (2011) and Kohlmann et al. (2007, 2010) studies have concluded that it is the cloud forest, which is the most biodiverse and endemic-rich environment. The inclusion of these two new scarab-beetle species into the count continues to support the previous findings.

As mentioned previously in another paper (Solís and Kohlmann 2012), a mitochondrial DNA analysis of Costa Rican scarabaeines is being undertaken. Partial results are presented here, analyzing the relationship between the *Onthophagus dicranius* Bates species-group (Kohlmann and Solís 2001) and the *Onthophagus clypeatus* Blanchard species-group (Zunino and Halffter 1997).

## Materials and methods

Specimens studied came from the insect collection of the Museo Nacional de Costa Rica (National Museum of Costa Rica, ex INBio collection). All type material (holo- and paratypes) of both species is deposited in the same collection.

The specimens were studied using a Zeiss Stemi 2000–C stereozoom binocular microscope. Measurements were made to the nearest 0.1 mm using an ocular micrometer. Morphological nomenclature follows Kohlmann and Solís (2001) and Solís and Kohlmann (2013).

The synthetic aperture radar (SAR) image of Costa Rica, which has been used as the base map in figures 5 and 6, was downloaded from the NASA website (https://www2.jpl.nasa.gov/srtm/central_america_radar_images.html). The maps were made and edited using the QGIS geographic information system software; this program is open source on the Internet for multiple platforms (https://qgis.org/en/site/forusers/download.html).

We downloaded from the Internet publicly available raster-type bathymetric maps, obtained from the General Bathymetric Chart of the Oceans (GEBCO) website (https://www.gebco.net/data_and_products/gridded_bathymetry_data/). We also obtained raster elevation maps freely available from the NASA project website and the Ministry of Economy, Trade and Industry (METI) of Japan, called the Advanced Spaceborne Thermal Emission and Reflection Radiometer (ASTER), Global Digital Elevation Model (GDEM) (https://asterweb.jpl.nasa.gov). In addition, we used the commercial program Photoshop CS3 Extended Version 10.0.1 to prepare the versions for this publication.

The mitochondrial DNA information (DNA barcoding) was obtained through the methodology described by Wilson (2012) and accessible through the Barcode Data System (BOLD), the cloud-based data storage and analysis platform developed at the Center for Biodiversity Genomics in Canada (http://boldsystems.org).

## Taxonomy

### Onthophagus humboldti sp. nov.

Taxon classificationAnimaliaColeopteraScarabaeidae

4E574931-E272-5135-B5A6-89A4E7DD70E0

http://zoobank.org/C13D9441-5A22-4CA1-974F-88B6A97199AC

**Figure 1.**
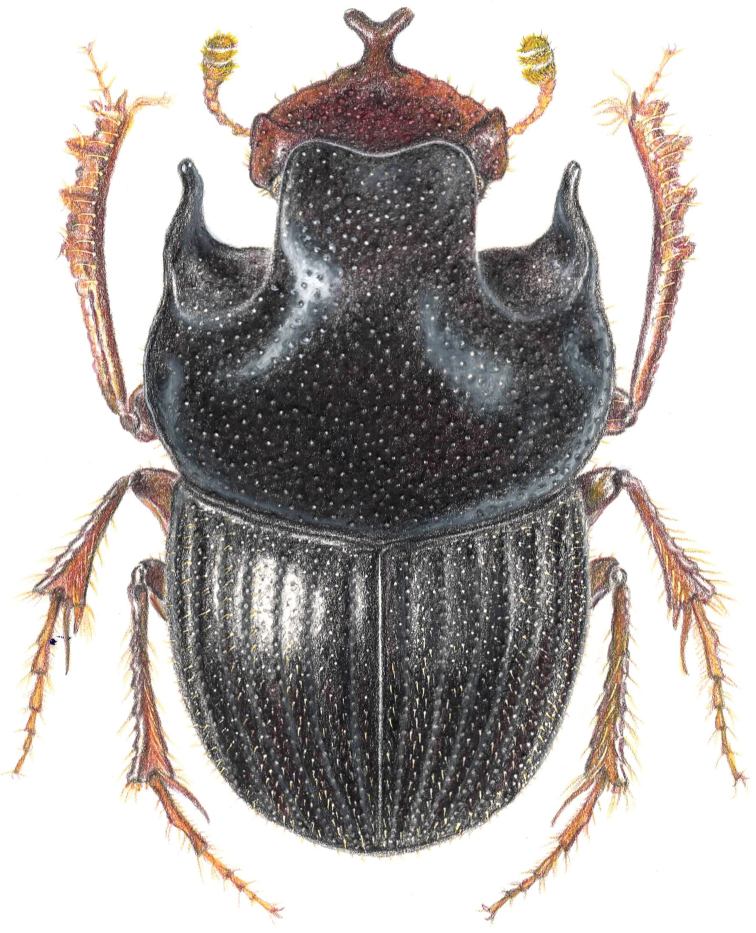
Dorsal drawing of a male *Onthophagus humboldti* sp. nov.

**Figure 2.**
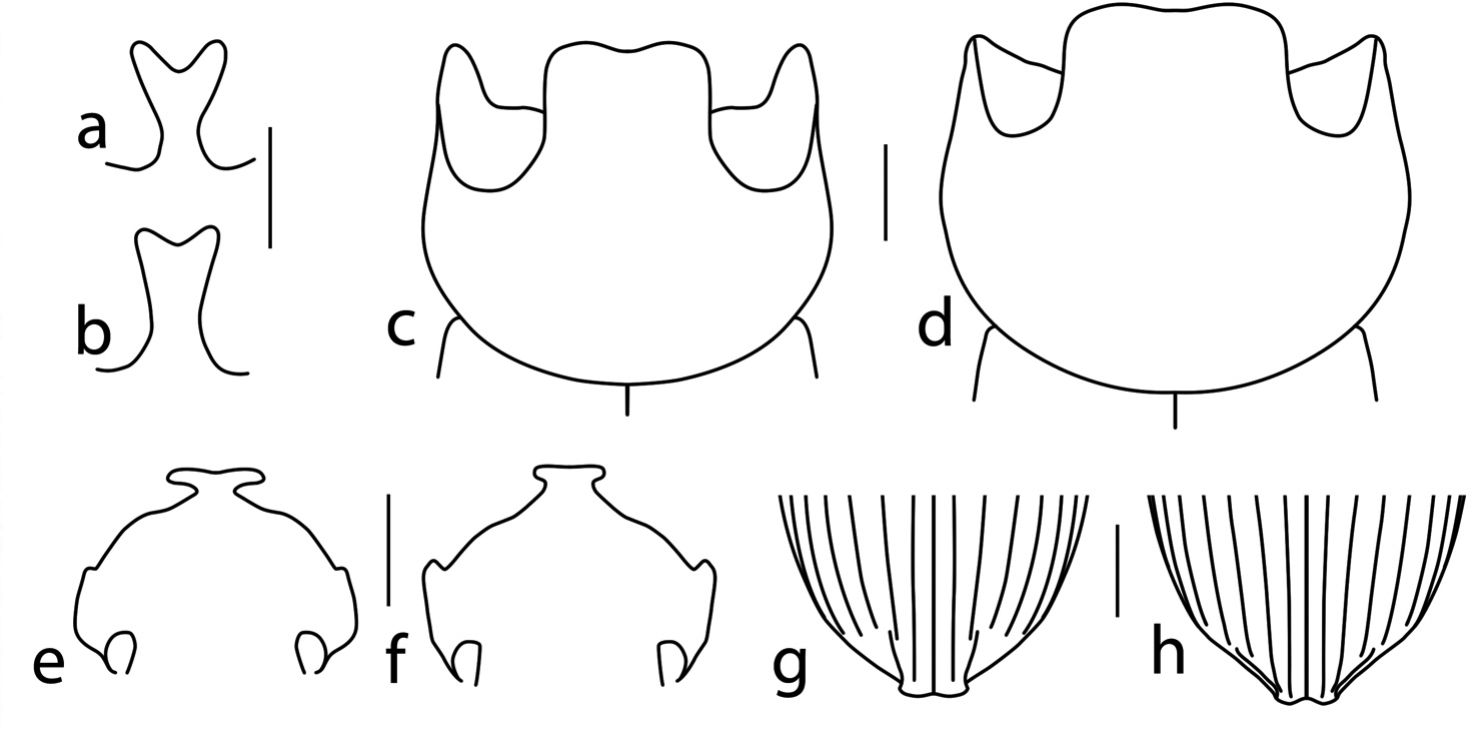
Drawings of the clypeal horn of **a***O. humboldti* sp. nov. and **b***O. micropterus*; pronotum of **c***O. humboldti* sp. nov. and **d***O. micropterus*; head of **e***O. humboldti* sp. nov. and **f***O. micropterus*; and elytral apex of **g***U. bonplandi* sp. nov. and **h***U. dybasi*. Scale bars: 1 mm.

**Figure 3.**
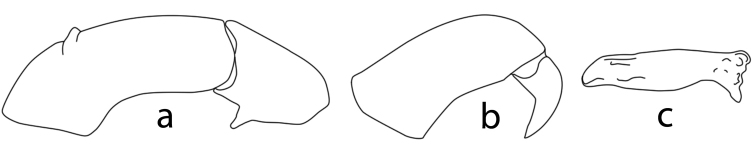
Aedeagui of **a***O. humboldti* sp. nov. **b***U. bonplandi* sp. nov. **c** brachypterous wing of *O. humboldti* sp. nov.

Figures 1 , 2a, c, e , 3a, c , 5 , 6 , 7 , 9

#### Type locality.

Costa Rica. Prov. Puntarenas. Buenos Aires, P.N. La Amistad. Tres Colinas.

#### Type deposition.

Museo Nacional de Costa Rica, Santo Domingo de Heredia, Costa Rica.

#### Type material.

Holotype male, pinned, with genitalia in a separate microvial. Original label: “Costa Rica. Provincia Puntarenas. Buenos Aires, Parque Nacional La Amistad. Tres Colinas. 2100–2200 m. 27–29 Febrero 2008. A. Solís, M. Moraga. Trampa Foso. L S 343850 565700.” “HOLOTYPE/Onthophagus humboldti Kohlmann, Solís, Alvarado [red printed label]”.

#### Other material.

Paratypes. (8 males, 4 females). “Costa Rica. Provincia Puntarenas. Buenos Aires, Parque Nacional La Amistad. Tres Colinas. 2100–2200 m. 27–29 Febrero 2008. A. Solís, M. Moraga. Trampa Foso. L S 343850 565700.”

#### Diagnosis.

Elytra as long as or shorter than pronotum (Fig. 1), due to brachyptery (Fig. 3c). Broad clypeal horn bifurcation (Fig. 2a); pygidium and apex of elytra with evident setae; clypeal margin indented at junction with clypeo-genal suture (Fig. 2e).

#### Description.

***Holotype.*** Male (Fig. 1), length 7.2 mm; maximum width 4.3 mm. Oval, shining reddish black. Centre of the clypeus projected forming a slender bifid horn (Figs 1, 2c); genae projected forming an angle (Fig. 2e), genal sutures almost effaced; head punctures coarse, regular, dense, becoming finer and sparser towards the center; clypeal carina absent, vertex carina substituted by two vertical asymmetric platelets, modestly developed, and obliquely oriented; eyes two times longer than wide and separated by eight times the eye width.

Pronotum (Fig. 2c) very convex, lateral margins with a small and irregular fovea, not lineal; lateral pronotal margins bordered by a deep sulcus, anterior and basal borders margined; pronotal surface reticulate and covered by dense, regular, coarse, annular, and deep punctures without setae; pronotal projection well-developed (Figs 1, 2c), forming a broad bilobed plate slightly bent downwards, with a depressed area antero-centrally, and having clear antero-lateral margins; anterior angles projected as long, slender, and curved projections (Figs 1, 2c); pronotal base with a sulcus extending forward one third its length; scutellum not visible between the base of the elytra.

Elytra convex, with clear margins and without a humeral callus; with eight well-marked striae, fine and clearly impressed and with crenulating punctures; intervals clearly punctured, punctures big and dense, not aligned, bearing short, stiff setae along the lateral and apical margins; microsculpture reticulate and regular. Wing brachypterous, measuring 0.75 mm (Fig. 3c). Pygidium moderately shiny and shagreen, margined border, with big, coarse, annular punctures bearing short and stiff setae. Aedeagus as Fig. 3a.

Mesosternum with evident annular punctures bearing no setae. Metasternum shagreen and finely punctured, more coarsely laterally, basal third with a sulcus. Abdominal segments shagreen and finely punctured.

Fore femur long, slender, and punctured; meso- and metafemur short and elongate, light yellow. Fore tibia long, slender and arched (Fig. 1); with four external teeth; tibial spur elongated, straight, pointed, deflexed anteriorly, extending to second tarsal segment. Middle- and hind femur light yellow at middle.

Female, length 6.3 mm; maximum width 3.6 mm. It is similar to the male and varies in having a clypeus not forming a horn, clypeus shagreen, genae not projected as teeth, with a head frons keel, two small platelet projections at head vertex, no pronotal projection, no projected pronotal anterior angles, fore tibia short, fore femur short, last abdominal sternite broad.

#### Variation.

Length 5.6 to 7.2 mm. Width 3.2 to 4.3 mm. Small males do not have the bifid clypeal horn, just a small erect lamella; vertex platelets forming a small projection; anterior pronotal angles not projected, pronotal projection forming a small carina. Body color varying from black to piceous red.

#### Etymology.

This species is dedicated in honor of Friedrich Wilhelm Heinrich Alexander von Humboldt, Prussian geographer, explorer, and naturalist, commemorating the 250^th^ anniversary of his birth. He is widely recognized for fathering the work on physical and plant geography, which laid the foundation for the development of modern biogeography.

#### Taxonomic considerations.

Kohlmann and Solís (2001) report the existence of 39 species of *Onthophagus* for Costa Rica. This new species would increase their numbers to 40. *Onthophagus humboldti* sp. nov. belongs to the *Onthophagus dicranius* Bates species group, as defined by Kohlmann and Solís (2001).

*Onthophagus humboldti* sp. nov. will key out to *O. micropterus* Zunino & Halffter, 1981, in Kohlmann and Solís´ key (2001). It can be easily differentiated by the following characteristics: In males clypeal horn slender at middle and very bifurcated at apex (Fig. 2a) (*O. humboldti* sp. nov.) versus broad at middle and notched at apex (Fig. 2b) (*O. micropterus*); genae projected forming an angle (Fig. 2e) (*O. humboldti* sp. nov.) versus genae projected forming a tooth (Fig. 2f) (*O. micropterus*); vertex platelets forming a carina (*O. humboldti* sp. nov.) versus a pointed projection (*O. micropterus*); anterior lateral angles of pronotum projected as long, slender, and curved projections (Fig. 2c) (*O. humboldti* sp. nov.) versus a short, curved projection (Fig. 2d) (*O. micropterus*); pronotal central forward projection well-developed, forming a broad bilobed plate slightly bent downwards (Fig. 2c) (*O. humboldti* sp. nov.) versus a bilobed plate projecting forward (Fig. 2d) (*O. micropterus*). In females: vertex platelets forming a carina (*O. humboldti* sp. nov.) versus a pointed projection (*O. micropterus*).

#### Geographical distribution.

This species is so far only known from the area of Tres Colinas, near Buenos Aires, in the province of Puntarenas (Fig. 5). It has been collected from 2100 to 2200 m altitude in the month of February in lower montane rain forest.

#### Chorological affinities.

*Onthophagus humboldti* sp. nov. is endemic to the Cordillera de Talamanca and is the tenth known brachypterous *Onthophagus* species to be described worldwide. A closely related species, *O. micropterus*, is also distributed in the Cordillera de Talamanca (Fig. 6), from 2100 to 3000 m altitude in tropical mountain rainforest and has been collected from October to February.

#### Biogeography.

This species belongs to the *O. dicranius* species group, as established by Kohlmann and Solís (2001). This group of species has extra-American affinities, in which Howden and Gill (1993) indicate that the American fauna of *Onthophagus* is the result of invasive species from East Asia and that the *O. dicranius* group presents characters in common with New Guinea species. This agrees with the hypothesis originally proposed by Zunino and Halffter (1988), which points out for the supraspecific groups of American *Onthophagus*, an origin of its lineages, which in the case of the current representatives is distributed in East or Southeast Asia; and for this case, the Asian representation of the ancestral line, like the American one, has its distribution present in the humid tropics. On the other hand, the *O. dicranius* species group has its present-day center of diversity in tropical North America and relatives in South America (Zunino and Halffter 1997; Kohlmann and Solís 2001).

This situation seems to be in congruence with the boreotropical distribution hypothesis (Wang 1961; Wolfe 1975; Lavin and Luckow 1993; Xiang and Soltis 2001; Davis et al. 2002), where current flora groups show a tropical disjunct distribution, generally centered in America, Africa, and tropical Asia. This hypothesis is based on the observation of the existence of tropical broadleaf forests during the Early Paleogene (in old Stratigraphy terminology, Early Tertiary) at high latitudes in regions that are currently temperate, directed by a Late Paleocene-Early Eocene thermal maxima (ca. 52 ma, Zachos et al. 2001) and that many current angiosperm temperate taxa have evergreen relatives in subtropical rainforests (Axelrod 1966). This proposal then suggests the existence of northern bridges that were once at lower latitudes, such as the Bering Bridge during the Early Paleogene and the North Atlantic Bridge during the Eocene, which may have served as migration routes for groups of organisms that currently present intercontinental disjunct distributions. This hypothesis suggests that a taxon with a present-day center of diversity in tropical North America, and with an early Paleogene fossil record from any region there, has a high probability of having sister-group relatives in the Paleotropics and derived relatives in South America (Lavin and Luckow 1993).

This pattern of distribution would clarify those proposed by Halffter (Halffter and Morrone 2017) for the “Mexican Transition Zone” in particular one of them, the so-called “Paleoamerican Dispersion Pattern” (Halffter 1964). This pattern of dispersion corresponds to northern taxa that arrived in North America from Eurasia, and has been subdivided by Halffter et al. (1995) into four variants, where one of them, called the “Paleoamerican Tropical Pattern”, corresponds to species found in the lowlands of the tropics and at medium altitudes, their distribution being very similar to that of the Neotropical pattern, but their affinities are with the Old World taxa. Halffter et al. (1995, 2008) placed the *Onthophagus clypeatus* and *>Onthophagus dicranius* species groups of the genus *Onthophagus* within this pattern.

Actually, the groups of species mentioned above are congruent with the typical characteristics of the so-called boreotropical distribution. Therefore, the aforementioned distribution variant, the “Paleoamerican Tropical Pattern”, seems to be the same with the boreotropical distribution and it is proposed here to use the term boreotropical distribution from now on as it is a more complete and well-founded concept, besides being an older one. This pattern has been studied and characterized at very fine phylogenetic and biogeographic analysis levels in animal and plants (Lidgard and Crane 1990; Xiang and Soltis 2001; Davis et al. 2002; Feng et al. 2009; Guo et al. 2012; Ye et al. 2016).

### Uroxys bonplandi sp. nov.

Taxon classificationAnimaliaColeopteraScarabaeidae

CD2B9D8B-870A-5AA7-87E6-7D6936B3E89F

http://zoobank.org/E8FB3E6C-6E3B-4C5E-9550-6A4238DD70EB

**Figure 4.**
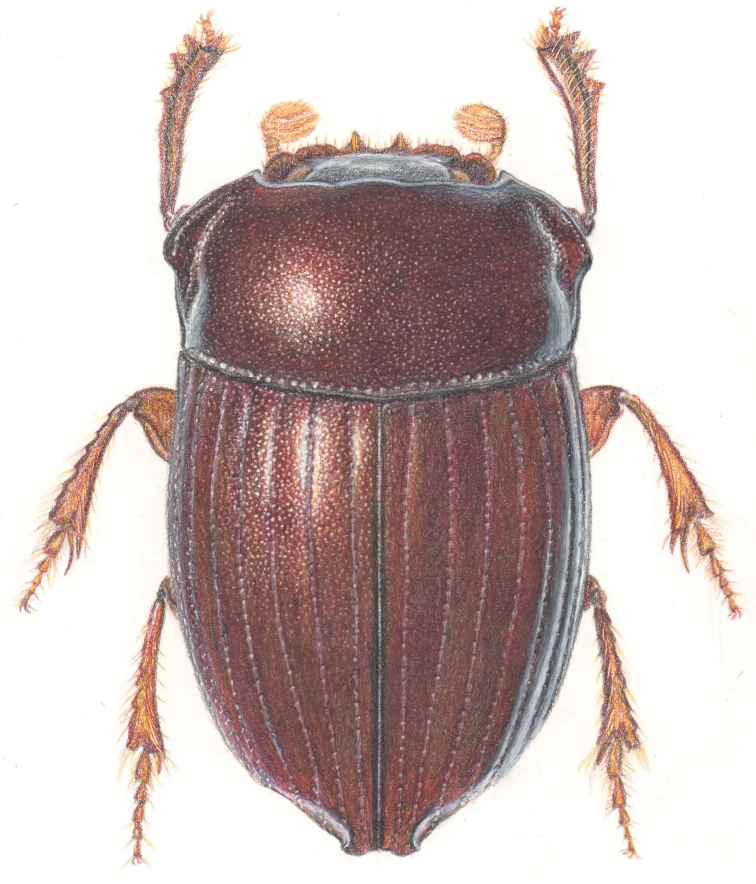
Dorsal drawing of a male *Uroxys bonplandi* sp. nov.

**Figure 5.**
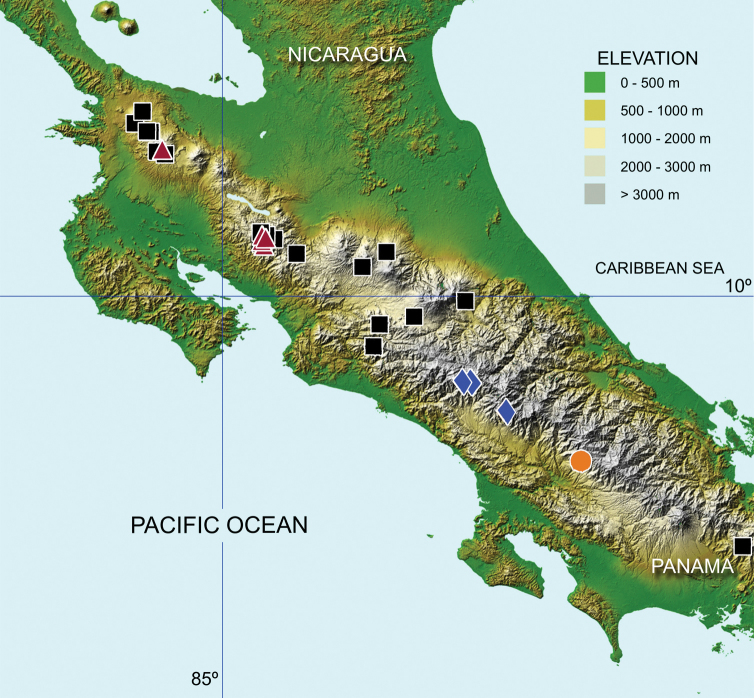
Known distribution of *O. humboldti* sp. nov. (orange circle) and *U. bonplandi* sp. nov. (red triangle). The distribution of the proposed sister species of these new taxa is also depicted, *O. micropterus* (blue rhombus) and *U. dybasi* (black square).

Figures 2h , 3b , 4 , 5

#### Type locality.

**Costa Rica.** Guanacaste. Sector Santa María, path to the cone of the Santa María, part of the Rincón de la Vieja volcanic massif, 1565 m.

#### Type deposition.

Museo Nacional de Costa Rica, Santo Domingo de Heredia, Costa Rica.

#### Type material.

Holotype male, pinned, with genitalia in a separate microvial. Original label: “Costa Rica. Provincia Guanacaste. Sector Santa María, Sendero a Pico Volcán Santa María. 1565 m. 2 Diciembre 2017. Col. Sergio Salas Ríos. Biocol. 10.8039N, 85.3281W.” “HOLOTYPE/Uroxys bonplandi Kohlmann, Solís, Alvarado [red printed label]”.

#### Other material.

Paratypes (18 males, 25 females). “Costa Rica. *Provincia Guanacaste.* Sector Santa María, Sendero a Pico Volcán Santa María. 1565 m. 2 Diciembre 2017. Col. Sergio Salas Ríos. Biocol. 10.8039N, 85.3281W (6 males, 10 females). “Tilarán Bosque Nuboso Santa Elena. 1600 m. 26 Noviembre – 8 Diciembre 1999. J. Rodríguez Trampa de Luz. L N 258000 45000” (1 female). “*Provincia Puntarenas.* Monteverde Zona Protectora Arenal-Monteverde. Parcela Brillantes. 1500–1600 m. 17–19 Junio 2009. A. Solís, J.D. Gutiérres. Trampa Foso. L N 252009 450981” (4 males, 2 females), “13–1600 m. 10°18'N, 84°48'W. Univ. California EAP 1991” (1 female). “Est. La Casona. 1520 m. Reserva Biológica Monteverde. N. Obando. Octubre 1991. L N 253250 449700” (2 males, 2 females), “Septiembre 1990 (1 male), 29 Nov – 17 Diciembre 1994, K. Martínez, L N 253200 449700” (2 males, 1 female). “*Provincia Alajuela.* San Ramón. Zona Protectora Arenal-Monteverde. Parcela El Valle. 1600–1700 m. 16–18 Jun 2009. A. Solís, J.D. Gutiérrez. Trampa Foso. L N 255970 452538” (3 males, 9 females).

#### Diagnosis.

Anterior of frons evenly convex, without carina or groove, with a dimple or transversely rugose; clypeal margin indented at junction with clypeogenal suture; dorsal ocular area twice as long as wide, distance between eyes five times eye width; pronotum evenly convex, sides angled near middle; elytral apex of the second to fourth intervals forming an oblique keel (Fig. 2h); basal sulcus of pygidium sinuate; fore tibial spur slender and deflexed distally.

#### Description.

***Holotype.*** Male, length 7.4 mm; maximum width 3.8 mm. Elongate oval, shining reddish black (Fig. 4). Clypeus bidentate, slightly indented immediately laterad of teeth; teeth broadly triangular and strongly reflexed (Fig. 4). Head surface with a small dimple at the center, distinct small punctures throughout. Clypeogenal suture distinct; clypeal margin distinctly indented at intersection of suture (Fig. 4); genal margins broadly rounded (Fig. 4). Frons weakly convex, with very slight, broad indentations. Dorsal ocular areas approximately twice as long as wide at posterior edge of canthus (12 to 14 facets wide at that point), distance between ocular areas approximately five times their width.

Pronotum at median angulation as wide as elytra; lateral edges of pronotum produced into prominent angles (Fig. 4), strongly sinuate on lateral view, posterior two-thirds of margin nearly vertical; pronotum weakly convex medially; surface densely covered with fine, deep punctures; median longitudinal sulcus feebly indicated in posterior third; lateral fovea in form of crenulated longitudinal deep grove three-fourths length of pronotum (Fig. 4), not extending to either anterior or posterior margin, with cluster of coarse punctures in posterior third; pronotum margined basally, with adjacent row of large longitudinal punctures (Fig. 4).

Elytron moderately convex, clearly punctate (faintly in *Uroxys dybasi* Howden & Young, 1981), humeral umbone small; striae distinct but shallow, with distinct punctures evenly spaced for most of length of each stria, seventh stria extending three-fifths length of elytron; posterior tenth of first stria furrowed; intervals flat, slightly flattened and constricted, not produced, except at the apex of the second to fourth intervals forming an oblique keel (Fig. 2h) (sharp straight keel in the third interval in *dybasi*, Fig. 2g).

Meso- and metasternum clearly punctate (faintly in *dybasi*); meso-metasternal suture medially moderately angulate anteriorly, moderately angulate laterally, three times farther from anterior margin of mesosternum than from mesocoxal cavity; metasternum swollen, with distinct median posterior depression.

Ventral abdominal segments two to five of equal length medially, each only slightly shorter medially than sixth; sixth slightly longer laterally than medially; anterior margins with small punctures (big crenulated punctures in *dybasi*). Pygidium strongly convex, faintly punctate, twice as wide as long; sulcus surrounding disc deep basally, shallow elsewhere; margin formed of same width apically and laterally; sulcus basally very slightly arcuate toward apex on each side of midline.

Fore tibia elongate with inner margin broadly curved (Fig. 4); outer margin with three teeth in apical third, teeth approximately equidistant, basal tooth somewhat reduced and more broadly triangular (Fig. 4); apex of fore tibia with short, narrow, rounded, deflexed projection at inner corner, projection approximately half length of tibial spur. Tibial spur elongated, straight, pointed, extending to fourth tarsal segment. Fore femur gradually tapering distally; middle femur with a faint ventral posterior triangular projection at apical third (evident projection in *dybasi*); hind femur with a well-developed ventral posterior swelling at apical third; posterior margin of hind trochanter continuous with posterior margin of femur.

Female, length 6.9 mm; maximum width 3.6 mm. It is similar to the male and varies in having a rugose clypeus, lateral edges of pronotum produced into less prominent angles. Elytral apex without oblique keels. Fore femur and fore tibia not as long. Middle and hind femur without a projection or swelling at apical third.

#### Variation.

Length 5.7 to 7.6 mm. Width 3.2 to 4.1 mm. The center of the head might have a small dimple and/or also a slight transverse rugosity.

#### Etymology.

This species is dedicated in honor of Aimé Jacques Alexandre Goujaud Bonpland, French naturalist, physician, and botanist, member of the scientific expedition that accompanied Humboldt to Spanish America.

#### Taxonomic considerations.

Solís and Kohlmann (2013) report the existence of 12 species of *Uroxys* for Costa Rica. This new species would increase their numbers to 13. Due to its great similarity, we here propose that *Uroxys bonplandi* sp. nov. represents the sister species of *U. dybasi* Howden & Young, 1981.

*Uroxys bonplandi* sp. nov. will key out to *U. dybasi* in Solís and Kohlmann´s (2013) key. It can be easily differentiated by the following characteristics: *Uroxys bonplandi* sp. nov. is consistently bigger (5.7 to 7.6 mm) than its sister species (4.3 to 5.6 mm), *U. dybasi*. It can also be separated by the clear punctures in thorax and elytra in *bonplandi* sp. nov. (faint in *dybasi*). In males: elytral apex of the second to fourth intervals forming an oblique keel in *bonplandi* sp. nov. (Fig. 2h) (sharp straight keel in the third interval in *dybasi*, Fig. 2g), meso- and metasternum clearly punctate on *bonplandi* sp. nov. (faintly in *dybasi*), anterior margins of ventral abdominal segments with small punctures in *bonplandi* sp. nov. (big crenulated punctures in *dybasi*), and middle femur with a faint ventral posterior triangular projection at apical third in *bonplandi* sp. nov. (evident projection in *dybasi*).

#### Geographical distribution.

*Uroxys bonplandi* sp. nov. has been collected so far in the Cordillera de Guanacaste and the Cordillera de Tilarán (Fig. 5). It is a mountain species distributed from 1520 to 2200 m of altitude and has been collected in the following life-zones: wet tropical forest (premontane transition), lower montane rain forest, lower montane wet forest, premontane rainforest, and premontane wet forest. It has been collected from June to February.

#### Chorological affinities.

*Uroxys bonplandi* sp. nov. coincides with *U. dybasi* in being distributed along the Guanacaste and Tilarán mountain ranges. (Fig. 5) This last species has been also reported from mountain forests from Panama in the Cordillera de Chiriquí and in Costa Rica in the Cordillera Central and Talamanca (Fig. 5), being distributed between 600 and 1700 m and collected throughout the whole year. *U. bonplandi* sp. nov. represents also the first known endemic species of *Uroxys* for Costa Rica.

Another related species is *Uroxys tacanensis* Delgado & Kohlmann, 2007, known only from its type locality, the Tacaná volcano, at the border of Mexico and Guatemala, living in cloud forest at 2000 m altitude (Delgado and Kohlmann 2007). No other species of this group has been yet collected in the intermediate areas. They are all montane species.

## Discussion

### Brachyptery

As mentioned above, *O. humboldti* sp. nov. is a brachypterous species. There are also another two brachypterous species of *Onthophagus* in Costa Rica: *O. inediapterus* Kohlmann & Solís, 2001 (*Onthophagus dicranius* Bates line) and *O. micropterus* Zunino & Halffter, 1981 (*Onthopagus dicranius* Bates line) (Kohlmann and Solís 2006). Other six brachypterous species are also known for Mexico: *O. brachypterus* Zunino & Halffter, 1997 (*O. landolti* Harold group); *O. chilapensis* Gasca-Álvarez, Zunino & Deloya, 2018 (*O. chevrolati* Harold group); *O. gilli* Delgado & Howden, 2000 (*O. chevrolati* Harold group); *O. inflaticollis* Bates, 1886–1889 (*O. chevrolati* Harold group); *O. pedester* Howden & Génier, 2004 (*O. landolti* Harold group); and *O. zapotecus* Zunino & Halffter, 1988 (*O. landolti* Harold group) (Kohlmann and Solís 2006; Gasca-Álvarez 2018). All the aforementioned species live in areas of old geological emergence: Cordillera de Talamanca and Sierra Madre del Sur (Kohlmann and Solís 2006). Outside the American continent there is only one brachypterous *Onthophagus* species known from Australia, living in vine scrub in arid areas, *O. apterus* Matthews, 1972 (Matthews 1972). All the American species have in common that they inhabit humid montane forests, a habitat considered as stable by most ecologists. It is interesting to note that *Onthophagus* brachyptery in Costa Rica is confined to the *Onthophagus dicranius* line, whereas in Mexico it is confined to the *O. landolti* and *O. chevrolati* species lines. This would suggest a line independent morphological convergence to similar ecological/historical conditions.

In relation to wing reduction, Zunino and Halffter (1988) described the most extreme case then known in Onthophagini with the example of *O. zapotecus*, where the wing does not show any trace of venation and the wing length to body length ratio has a value of 0.156. On the other hand, *O. micropterus* presents a ratio of 0.205 (Zunino and Halffter 1981), whereas *O. humboldti* sp. nov. has a ratio of 0.107. This ratio represents at present the most extreme case known so far of wing reduction in onthophagine scarab beetles. The wing does not show any trace of wing venation. The elytra are not fused together, but are strongly interlocked. This species shows also narrowed elytral humeri, as well as shortened elytra as had been already observed by Darlington (1936) for carabid beetles and Scholtz (2000) for scarab beetles. Contrary to the observation made by Scholtz (2000) and Scholtz et al. (2009) that flightless scarabs have reduced eyes with a smooth margin, *O. humboldti* has no such condition; however, as indicated by Scholtz et al. (2009), this species has a rounded body shape.

**Figure 6.**
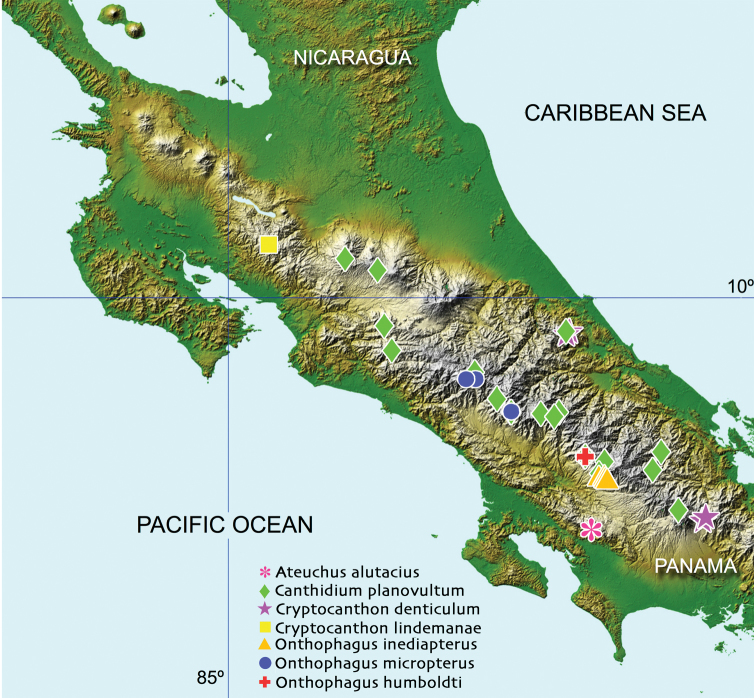
Known distribution of brachypterous Scarabaeinae in Costa Rica.

Accepted wisdom has proposed that in Scarabaeoidea the evolution of flightlessness is related to temperate highland forests in the tropics; arid environments, such as deserts; temperate forests at low latitudes in the southern hemisphere; islands; termite nests; and cold regions (Zunino and Halffter 1988; Génier 2000; Scholtz 2000). In montane environments, where *Onthophagus*, and other brachypterous genera, such as *Ateuchus*, *Canthidium*, and *Cryptocanthon* live in Costa Rica (Fig. 6), flight is apparently non-essential. According to Darwin´s classical explanation (Darwin 1859), known as “Darwin´s factor” (Darlington 1971), the presence of strong mountain winds could drag flying individuals towards unfavourable habitats for their survival (Zunino and Halffter 1981, 1988). This last explanation is strongly contested by Roff (1990), because it does not correctly take into account scale issues.

Scholtz et al. (2009) propose that flightlessness increases in scarab beetles with altitude in temperate forests in the tropics (which are considered to be a stable habitat), being rare in lowland tropical forests. A similar process has been observed in carabids, where brachypterous species also predominate in montane areas (Darlington 1971); as well with passalid beetles in tropical humid montane forests (Mac Vean and Schuster 1981). This seems to be the case for the present study, where all brachypterous species are montane, no species having been found in the lowlands so far. This would support Roff’s (1990) original hypothesis that flightlessness increases with altitude.

Wagner and Liebherr (1992) present an analysis of flightlessness in insects, indicating that around 10 % of temperate Coleoptera show this characteristic. Based on our current tally, 184 Scarabaeinae taxa have been listed for Costa Rica; of these, seven species are brachypterous (Fig. 6), thus representing 3.8 % of flightless scarab beetles. The low brachyptery percentage found in Costa Rica would support the hypothesis that insect and scarab-beetle flightlessness increases with latitude (Roff 1990; Scholtz et al. 2009).

At present, a very much accepted hypothesis that tries to explain the origin of this phenomenon is the one given by Lawrence et al. (1991), where they propose that wing brachyptery may have a selection value in insects that have adopted a sedentary, cryptic or a parasitic way of life, or that live in mountain, island or high latitude habitats. Another explanation for this situation, and the one we follow and expand here, is the one proposed by Kavanaugh (1985). Kavanaugh suggests that macroptery represents the ancestral (plesiotypic) condition among beetles and that brachyptery has evolved independently many times among Coleoptera and other pterygote insects. Such a widespread phenomenon requires explanation. Brachyptery is a major factor contributing to restricted distributions and it usually does not progress to a stage where the wing rudiment is actually absent (Kavanaugh 1985). However, Frolov (pers. comm., 2019) has observed that in Orphninae (Scarabaeidae) two genera are completely apterous. Brachyptery has also been suggested as a factor that promotes speciation (Mayr 1963; Hackman 1964).

It is clear that the distribution of brachypterous forms is not random, certain patterns are repeated. In North and Central America no brachypterous Scarabaeinae are known from the lowlands, alpine regions, or from rodent nests. They are only known from the mountains in Costa Rica, especially the Talamanca range and the Sierra Madre del Sur in Mexico. In all cases these flightless species live in humid montane forests, spanning an altitudinal distribution that goes from 1100 to 3000 m. Flightlessness in scarabaeines is confined so far in Mesoamerica to small-sized genera, like *Onthophagus* (9), *Canthidium* (4), *Cryptocanthon* (2), and *Ateuchus* (1), in descending order of known species number. On the other hand, brachyptery seems to be confined in South American Scarabaeinae to eight medium-sized species of the genus *Dichotomius*, out of 170 described taxa, where this condition has evolved independently, at least four times in this genus (Nunes and Vaz-de-Mello 2013, 2016). However, this genus does not show a clear attachment to a particular ecological environment, because the different brachypterous species have been collected ranging from tree sand dune habitats to riparian forests (Nunes and Vaz-de-Mello 2016). According to models developed by Roff (1994), a dominant brachyptery can spread if the cost of being macropterous and habitat stability are important. In other words, regarding the last point, habitat stability is a key factor favoring the loss of flight (Roff 1990, 1994).

If it is generally accepted that the occurrence of brachyptery reflects long-term stability of habitats (Roff 1990, 1994), then one could propose that the occurrence of such forms in particular geographical areas is an indication of long-term stability for these regions as well, especially if this pattern is repeated by different taxa in the same area. This train of thought has been used for recognizing areas of long-term occupation, such as glacial refugia in carabids by Lindroth (1979) in Scandinavia and by Kavanaugh (1985) in Canada.

If one would plot the geographical ranges of the fore mentioned brachypterous scarab-beetles on a map, coincident occurrence of such taxa is apparent. The pattern that emerges is one in which one particular area stands out, the Cordillera de Talamanca (Fig. 6). All four known brachypterous genera and six species are concentrated in this range. All other known areas boast one to two genera and species and would probably represent minor centers or areas of subsequent colonization. These data would strongly suggest that the Cordillera de Talamanca has served as a center for both long-term survival and differentiation in this group of beetles, acting as a stable area. The Cordillera de Talamanca is the highest mountainous area in Costa Rica, and the highest range in Central America, reaching 3820 m altitude, and thus acting as a possible built-in buffer for residents against sudden and dramatic climate change. If the climate were to change rapidly and drastically, montane species could be able to move a short distance up or down in elevation, tracking their required microclimate, whereas lowland organisms would have to move far greater distances north or south in order to achieve the same result.

Lachniet and Seltzer (2002) and Vázquez-Selem and Lachniet (2017) analyzed the effects of the last glaciation on the Cordillera de Talamanca and estimated that the last local glacial maximum (LLGM) for the Cerro Chirripó occurred at 21–18 ka with a depression of the equilibrium line altitude (ELA) or snow line of ~1500 m in relation to the modern regional ELA of 4900–5100 m, thus representing a LLGM temperature reduction on the order of ~8–9 °C. However, a more recent research reevaluation by Potter et al. (2019) estimates the age of the LLGM at 25–23 ka for the Cerro Chirripó with a reconstructed ELA depression of 1317–1536 m and an associated cooling of ~7–9 °C. Palinological studies have indicated that during the last glacier interval (50–15.6 ka) with temperatures 7–8 °C cooler than today the treeless páramo extended down to 2100 m altitude, whereas it is distributed from 3300 to 3819 m at present (Islebe and Hooghiemstra 1997; Horn 2007). At the end of the last deglaciation (15.6–13 ka), the upper forest limit rose to 2700–2800 m (3100–3 300 m present-day forest limit of subalpine tropical rain/elfin cloud forest), indicating a temperature increase of up to 4.6 °C (Islebe and Hooghiemstra 1997; Horn 2007). Subsequently, the upper forest limit dropped 300–400 m from 13.1 to 11.2 ka indicating a temperature decline of 2–3 °C (Horn 2007). From 12.3 to 11.2 ka the glaciers retreated above 3500 m and the subalpine tropical rain forest was gradually replaced by mountain rain forest as the forest limit and temperatures rose toward present-day values (Islebe and Hooghiemstra 1997; Horn 2007).

**Figure 7.**
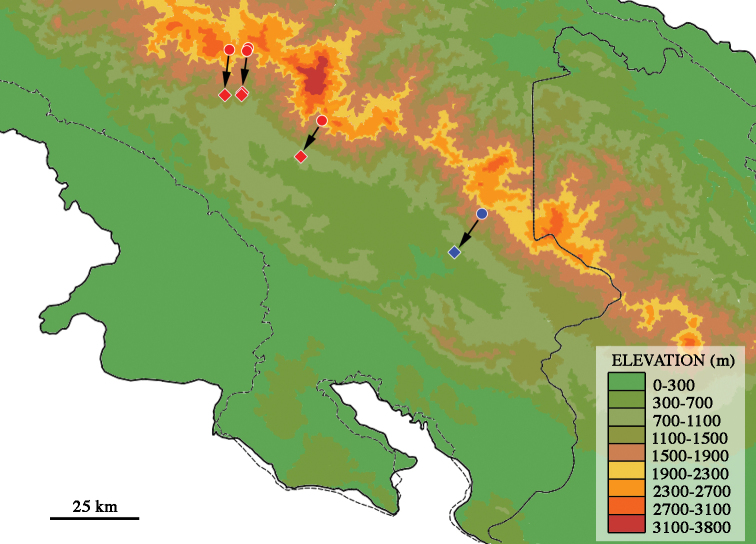
Present day distribution of *Onthophagus humboldti* sp. nov. (blue dot) and *O. micropterus* (red dot) and lines indicating proposed localities (rhombi) depressed by 1500 m (14 km in straight line) generated by the last glacial maximum, ~25–23 ka, in the Cordillera de Talamanca. All mountain systems are 150 m lower than present day height and an estimated sea level descent of 120 m is depicted. Dotted black lines represent present-day sea levels.

Figure 7 shows the present-day distribution of *Onthophagus humboldti* sp. nov. and *O. micropterus* and lines indicate proposed localities depressed by 1500 m (14 km in straight line) generated by the last glacial maximum, ~25–23 ka, in the Talamanca Cordillera. All mountain systems are depicted 150 m lower than present day height, considering a generalized continental uplift of 1 mm/year and an estimated sea level descent, due to glaciation, of 120 m. Interestingly, the glacially depressed localities are not only to be found at the base of the mountain system but also within the Valle de El General (Valley of the General or The General’s Valley), surrounded and probably climatically protected by the embracement of this very long valley (Kohlmann et al. 2002), that possibly dampened the cooling effect of the glaciation. Solano Quintero and Villalobos Flores (2001) did a climatic-geographic regionalization of Costa Rica and they found this intermontane valley to differ from the rest of the southern Pacific area in relation to having very homogeneous lower rainfall values (3050 m total annual precipitation) and a longer dry period (three months).

We propose here that the Valle de El General might have acted as a refugium for the brachypterous species during the last glacial maximum (LGM). This valley would then resemble what has been called a cryptic refugium (Stewart and Lister 2001; Stewart et al. 2010), a refugium situated at different latitudes or longitudes than would normally be expected, often resembling climatic islands in which conditions differ favourably from the surrounding areas. According to the classification proposed by Stewart et al. (2010), the Valle de El General would fit the description of being a glacial southern refugium, representing an accepted low-latitude refugium for temperate species during a glacial phase. Stewart et al. (2010) also indicate that one characteristic of cryptic northern refugia is that they are sheltered in habitats located in deeply incised valleys that provide microclimates for temperate species, which is precisely the case under study here. Stewart et al. (2010) do not present any examples in their study of cryptic glacial southern refugia (only cryptic interglacial southern refugia), so that the example of the Valle de El General could represent the first one reported of its kind. Finally, because the Valle de El General represents a small area (~1850 km^2^), and in accordance with island biogeography tenets (MacArthur and Wilson 1967) that indicate that because of low population size and a limited food base in small areas, this could explain that mostly small-sized (brachypterous and non-brachypterous) scarab species and not big-sized ones, could have had patches of suitable habitats to live in during glacial periods which seems to be the case for the present study, all reported brachypterous scarab beetle species in Costa Rica are small-sized. Coincidentally, six of the known seven brachypterous scarab beetle species are also distributed around the Valle de El General. This area is occupied by montane forests, representing incredibly biodiverse and more species-rich environments than lowland tropical forests in Costa Rica (Kohlmann et al. 2007, 2010; Kohlmann 2011). This high concentration of brachypterous species in these montane forests contradicts Scholtz (2000) and Scholtz et al. (2009) proposal that relatively species-poor, environmentally stable habitats, lacking complex biotic interactions, like temperate forests on tropical mountains, contribute toward flightlessness. Costa Rican montane forests are decidedly species-rich and thus most probably also having complex biotic interactions.

The Valle de El General must have been formed by the uplift of the Cordillera de Talamanca and the Fila Costeña (Costeña range), a process that began about 7 million years ago and accelerated during the last 4 million years, triggered by the arrival of the Coco submarine range (an extinct volcanic range, also known as Cocos Ridge) in the Pacific and by the compression of the microplate of Panama in the Caribbean. The Arenal depression (tectonic graben) must have originated less than 2 million years ago, although there is no better estimate. While the Valle Central (Central Valley) is of a more recent formation and its age goes back to less than half a million years (Alvarado et al. 2007; Alvarado and Gans 2012; Alvarado and Cárdenes 2016). The absence of endemic brachypterous species in the Valle Central can be a product of never having being present or that they were exterminated due to the cataclysmic volcanism that occurred several times in the last 800 ka, the last large one 322 ka ago, with the formation of pyroclastic flows (pyroclastic density currents), which destroyed everything in hundreds of square kilometers (burning ash clouds with a temperature > 600 °C, pyroclastic deposits with thickness between 10 and > 80 m); the last major event of this kind occurred some 322 ka, covered an extension of at least 820 km^2^ in the Valle Central and neighboring areas, as well as a distance of up to 80 km from the eruptive source (Pérez et al. 2006; Alvarado and Gans 2012). The cataclysmic and paroxysmal volcanism of this type (pyroclastic density currents or ignimbrites) has been absent in the last few million years both in the Valle de El General and in the Arenal tectonic depression, where, coincidentally, the only other known endemic brachypterous species (*Cryptocanthon lindemanae*) that does not live around the Valle de El General Area is present.

### Endemicity

**Figure 8.**
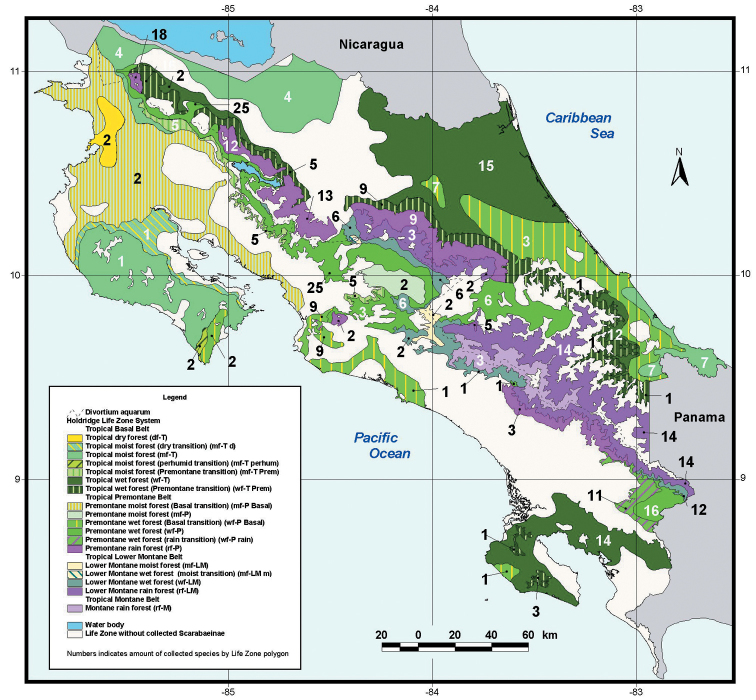
Map showing Costa Rican scarabaeine endemic numbers as distributed by life-zones (taken from Kohlmann et al. 2007).

Areas of endemism (AE) are fundamental areas in the analyses of biogeography and are defined as areas of non-random distributional congruence among taxa, whose biogeographical history probably shared common factors such as geological, ecological, or evolutionary processes (Harold and Mooi 1994; Morrone 2008). Important questions regarding the AE are its distribution and defining the major ecological/evolutionary factors (climatic/elevational gradients, geographic isolation, topographical heterogeneity) that affect the distribution of these areas. A quick glance at the distribution map of the new species and their closely related taxa (Fig. 5), as well as the map published by Kohlmann et al. (2007), showing the distribution of the endemic Costa Rican scarab beetle species (Fig. 8), shows a clear non-random distribution of the AE.

The mapping of the AE’s (Fig. 8) clearly identified mountain ranges as important centres of endemism. Endemism seems to be higher in the tropical mountains than in the tropical lowlands of Costa Rica. Similar results have been found in other areas of the world. Noroozi et al. (2018) have identified in the mountains of Iran using Asteraceae that patterns of endemic richness and areas of endemism identify mountain ranges as main centers of endemism, likely due to high environmental heterogeneity and strong geographic isolation among and within mountain ranges. Noroozi et al. (2018) also found that endemic richness and degree of endemicity are positively related to topographic complexity and elevational range. The proportion of endemic taxa at a certain altitude (percent endemism) is not congruent with the proportion of total surface area at this elevation, but is higher in mountain ranges. While the distribution of endemic richness (*i.e.*, number of endemic taxa) along an altitudinal gradient was hump-shaped peaking at mid-elevations, the percentage of endemism gradually increased with elevation. Sosa and Loera (2017) have shown that endemism of Mexican monocot geophytes was highest in montane regions (Mexican Trans-Volcanic Belt) and Millar et al. (2017) demonstrated that angiosperm endemism was highest in the mountains of New Zealand’s South Island. To very similar results came Buira et al. (2017) in relation to the vascular flora of the Iberian Peninsula. McDonald and Cowling (1995) found that endemic mountain fynbos flora (represented by shrubs with short-distance seed dispersal) are over-represented in high altitude wet habitats, where almost twice the number occur than expected on the basis of area occupied by these habitats. Finally, Scherson et al. (2014) found that coastal ranges in southern Chile have acted as glacial refugia for ancient flora during the Quaternary, showing a higher endemicity than expected by chance.

Noroozi et al. (2018) ask themselves the question, if endemics have higher numbers in the mountains than in the lowlands. This seems to be certainly the case in tropical Costa Rica for scarab-beetles (Scarabaeinae, Dynastinae) and monocot plants (Araceae, Arecaceae, Bromeliaceae) (Kohlmann et al. 2007, 2010; Kohlmann 2011), and as shown by other references, also around the world. High environmental heterogeneity and strong geographic isolation among and within mountain ranges seems to be a very plausible explanation. The effect of mountain systems as possible glacial refugia seems to also play an important role. So, as indicated in the previous section, because of simple geographical distance, mountains allow for small linear displacements that still maintain the same ecology, thus allowing a cenocron to stay concentrated in a small area; whereas, lowland species have to travel greater linear distances, hence presenting a much more extended distribution of endemic species.

### DNA mitochondrial analysis

An analysis of the cytochrome c-oxidase I (COI) for both new species was undertaken. The Bar Code Index Number (BIN) for each species is: *Onthophagus humboldti*, BOLD: ABA7524 and *Uroxys bonplandi*, BOLD: ABA3722. Results are clearly distinct, whereas the value registered for the *Onthophagus* pair gives an average Kimura–2–parameter [K2P] value of 6.35 % with a maximum distance of 10.6 %, the amount of DNA difference for the *Uroxys* pair is of only 3.3 %. *Uroxys* results stay in line with other similar ones calculated for a group of Caribbean-Pacific scarab-beetle sister-species pairs separated by the Cordillera de Talamanca which started its emergence around 7 million years ago; *Phanaeus pyrois* Bates, 1887 and *P. malyi* Arnaud, 2002 ([K2P]= 3.8 %) and *Phanaeus beltianus* Bates, 1887 and *P. changdiazi*Kohlmann and Solís 2001 ([K2P] = 3.0%), which show basically a similar amount of mitochondrial DNA difference (Solís and Kohlmann 2012). These average values are similar to the ones that Johns and Avise (1998) found (K2P difference) of 3.5 % in 47 pairs of bird sister species and divergences greater than 2% in 98% of vertebrate sister species.

However, the value shown for the *Onthophagus* sister-pair looks higher ([K2P] = 6.35 %). This is interesting if we consider that this pair is formed by flightless species and the geographical nearness between them, 52 km in a straight line (Fig. 5). It would seem therefore that a limited dispersal capacity tends to favour differentiation as Mayr had already suggested (1963). Another possible explanation is that this species pair represents an old clade, as is known that nucleotide substitutions accumulate through time (older clades tend to accumulate more substitutions).

This last explanation is concordant with the previous results shown in the brachyptery section, where it is suggested that the Cordillera de Talamanca has been an area of long-term stability, thus allowing the continuous and uninterrupted presence of clades. In general, areas with a preponderance of brachypterous populations represent areas of older populations (Lindroth 1979; Kavanaugh 1985). Volcanologically, the Cordillera de Talamanca and the Fila Costeña have been very stable during the last 9 and 3.5 million years, respectively. During these last 5 million years the Valle de El General starts to acquire its present geomorphological configuration due to the formation of the two aforementioned mountain systems (Alvarado and Gans 2012; Alvarado and Cárdenes 2016).

In a very interesting study of phylogenetics and biogeography of the genus *Onthophagus* inferred from mitochondrial genomes, Breeschoten et al. (2016) found that all New World species of *Onthophagus* form a monophyletic group. This study found an origin of the Onthophagini from an Afrotropical ancestral stock in the Eocene and a subsequent spread to the Americas via the oriental region at about 20–24 Ma. New World Onthophagini started diversifying around the Miocene (20 Ma).

Among the American Onthophagini that Breeschoten et al. (2016) studied, *O. clypeatus* Blanchard, 1843 from the tropical forests of Colombia to Bolivia (0–1000 m) and *O. rhinolophus* Harold, 1869 from tropical forests (0–1500 m) of Mexico to Guatemala, were included. They estimated a phylogenetic branching process between both of them at ca. 3 Ma (95 % HPD: 1.6–4.4 Ma). At that time and according to paleogeographic reconstructions of Alvarado and Cárdenes (2016), Costa Rica had already emerged. The Cordillera de Talamanca began to rise fast after 3.5 Ma with an estimated uplift rate of 1 mm/year (Miyamura 1975) and creating an uplifted area where montane species could start to evolve.

*Onthophagus clypeatus* Blanchard, 1846 and *O. rhinolophus* Harold, 1869, are part of the *clypeatus* species group and considered to be closely related to the *dicranius* species group (Zunino and Halffter 1997; Kohlmann and Solís 2001). These species are therefore related to *O. humboldti* sp. nov. and *O. micropterus*, and are also distributed in tropical mountain forests. This closeness might suggest a similar phylogenetic branching process of the Costa Rican species in the Cordillera de Talamanca around 3 Ma. One could suggest that after this relatively old speciation process the gradual evolution of flightlessness took place in a subsequent, much more recent time, where the orographic scenario was more akin to the present-day one. This scenario would be then in accordance with the aforementioned estimations of the rise of the Cordillera de Talamanca, as well as with the proposal of Sanmartín et al. (2001), where they found that in the western Nearctic animal diversification and animal species richness increased in the later part of the Neogene and early Quaternary (2.56 Ma), whereas they consider unlikely elevated speciation rates during the Pleistocene. However, Kohlmann et al. (2018) studying scarab beetles of the genus *Geotrupes* (Coleoptera: Geotrupidae), have found evidence that the last glacial maxima in the mountains of Oaxaca, Mexico (18–15 ka, but in Costa Rica around 24 ka), could explain very recent speciation processes, where speciation can be described as incipient, generating very closely related taxa with small taxonomic differences.

**Figure 9.**
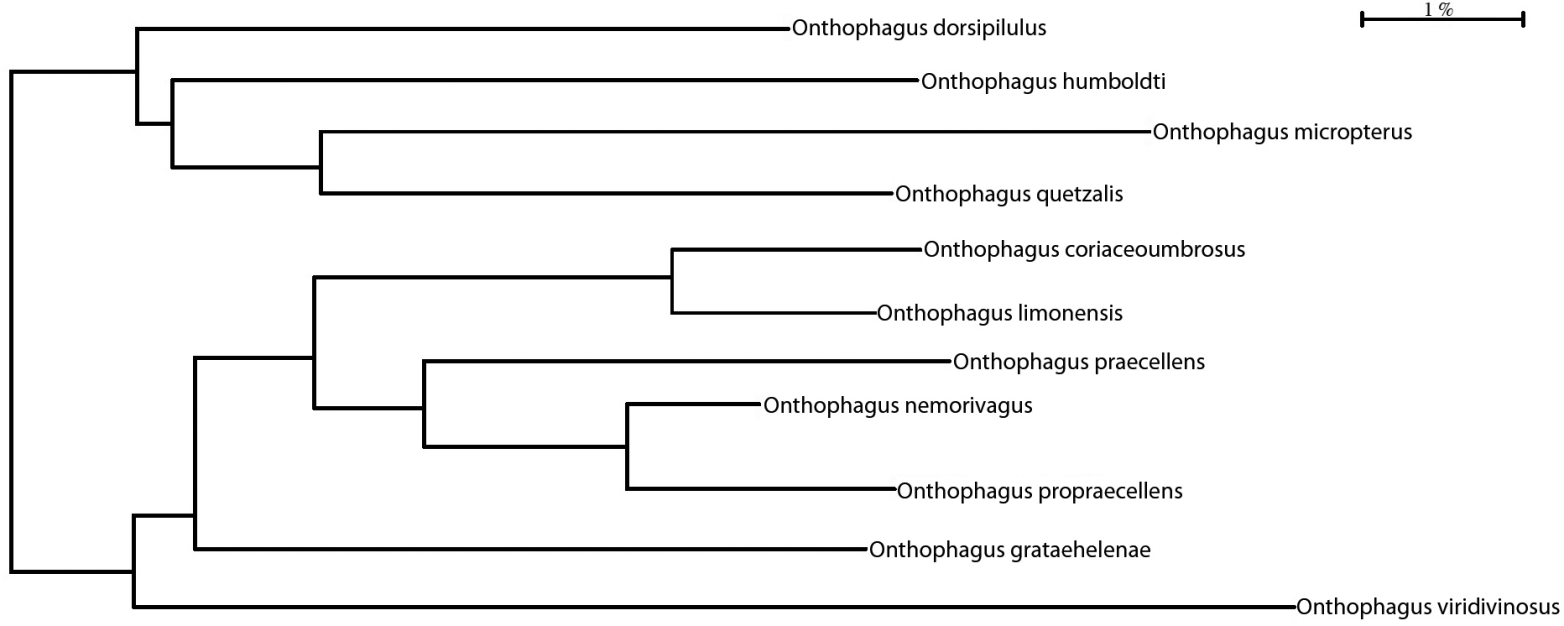
A cytochrome c-oxidase I (COI–5P) mitochondrial DNA sequence-based BOLD taxon ID tree of the nearest species of *O. humboldti* sp. nov.

We present in Figure 9 a cytochrome c-oxidase I (COI–5P) mitochondrial DNA sequence-based Bold Taxon ID tree of the species group to which *O. humboldti* sp. nov. belongs. The Taxon ID Tree is a functionality of the BOLD System, that allows for the generation of dendrograms from sequencing using the neighbor-joining algorithm.

BOLD uses neighbor-joining trees which group sequences together by the number of amino acid or nucleotide differences. The arrangement of the specimens in the tree is based on sequence similarities, with the sequences that are most similar placed closer together on the tree, and with the branch length indicating the degree of similarity. The percentage of similarity between sequences can be measured against the legend (line), where the longer the branch the more disparity between the sequences. It is often expected that specimens of the same species have more similar sequences and cluster closer together than specimens from different species.

This figure is part of a more general analysis done for the genus *Onthophagus* in Costa Rica. All four depicted taxa are mountain species distributed in the mountains of Costa Rica and Panama. *O. humboldti* sp. nov. seems to be a closely related species of *O. dorsipilulus* Howden & Gill, 1993, a species distributed in the Cordillera de Talamanca and the Cordillera de Chiriquí in Costa Rica and Panama from 1400 to 1800 m altitude and would seem to be its ecological equivalent at a slightly lower altitudinal belt. On the other hand, *O. micropterus*, also distributed in the Cordillera de Talamanca, seems to be the sister species of *O. quetzalis* Howden & Gill, 1993, a taxon distributed in the neighboring Cordillera de Tilarán and Guanacaste. The DNA mitochondrial analysis neatly recovers the formation of this cluster belonging to the *O. dicranius* species-group (Kohlmann and Solís 2001). The nearest species cluster to this last group is also included (Fig. 9), where all seven taxa belong to the *O. clypeatus* species-group (Zunino and Halffter 1997), as defined by Kohlmann and Solís (2001). The formation of these two well-defined, but closely related branches seems to support the proposal forwarded by Kohlmann and Solís (2001) that they are effectively two different groupings and not a single one, as proposed by Zunino and Halffter (1997).

Finally, *Onthophagus* having around 2200 valid species (Schoolmeesters 2016) and being a hyperdiverse and ecologically plastic genus, does not conform with Roff’s (1990) proposal that lineage size should favor the evolution of flightlessness. At present, only ten brachypterous species of *Onthophagus* are known: six in North America, three in Central America, and one in Australia. As a comparison, the genus *Dichotomius* Hope, 1838 has around 170 described species with eight brachypterous taxa (Nunes and Vaz-de-Mello 2013, 2016), all of them living in South America.

## Conclusions

The study of mountain biology retains all of its actuality and relevance. This study on tropical mountain brachyptery and endemicity falls in line with what Humboldt had already discovered (Humboldt 1805), which is the vertical progression of climate and vegetation in a mountain that explains the distribution and ecology of a species, as beautifully demonstrated by his drawing of plant distribution on the Chimborazo volcano published in his *Essai sur la géographie des plantes* (Humboldt 1805). This drawing can be considered as a veritable scientific epiphany in the case of Humboldt, so that it allowed him to connect all plant species according to their altitude and latitude. In other words, Humboldt could now establish the correlation between similar ecosystems in any part of the world. “Alles ist Wechselwirkung” (Everything is interaction) wrote Humboldt (1803) later in August 1803 in one of his diaries, while travelling in the Valley of Mexico. Everything is organically connected through multiple natural correlations. He could not have been more correct. This is Humboldt’s essence; he was far in advance of his time.

## Supplementary Material

XML Treatment for Onthophagus humboldti

XML Treatment for Uroxys bonplandi
